# Monitoring medication uptake in difficult asthma - qualitative analysis

**DOI:** 10.23889/ijpds.v1i1.349

**Published:** 2017-04-19

**Authors:** Debbie Anderson, Anne Boyter, Sean MacBride-Stewart, Douglas Cowan, Christine Bucknall

**Affiliations:** 1 Strathclyde Institute of Pharmacy and Biomedical Sciences; 2 NHS Greater Glasgow & Clyde

## Objectives

To determine the acceptability of using data on medicines dispensed in primary care to inform out-patient treatment of patients with difficult-to-treat asthma.

## Approach

Consultant respiratory physicians' access to a summary of all relevant medicines dispensed by community pharmacists to patients with difficult to treat asthma was piloted in 2015 (therapy review (TR)). Dispensed medicine data were collected using the patient’s unique NHS identifier. This information was aggregated monthly for the year before the patient attended their clinic appointment. Patients gave consent and the summary data were used to assess concordance with therapy and inform a discussion about future management.

Semi-structured interviews were conducted with eight patients who had received TR and eight respiratory physicians: two with access to the summary. The interviews aimed to highlight

- the experiences of patients and physicians on the utility of therapy reviews
- the views of physicians without access to summaries on the prospective use of therapy reviews.

With the participants consent, interviews were recorded and transcribed. Thematic analysis of grouped responses was conducted using NVivo software. 

## Results

All physicians agreed that poor compliance remains a significant concern when treating patients with difficult asthma and supported the use of TR. Physicians with experience of TR identified reliability over current methods of assessing compliance; ability to inform future treatment; and assistance in the discussion of concordance as advantages. The lag of three months in available dispensed data was a disadvantage. Physicians without experience of TR raised concern that use may lead to confrontation: reflected in the experience of one patient who expressed that TR discouraged them from improving compliance. Additional interventions are needed to improve compliance. Opinions from other patients were positive and supported the inclusion of TR as part of a consultation.

Physicians with experience of TR found the summary accessible, if access to computers containing specific software to view TR was available. This limitation was considered potentially problematic and physicians without access to TR expressed a preference to accessing TR via NHS Portal - a secure online platform permitting registered users access to patient-level information.

## Conclusion

This demonstrates the positive impact of using data about primary care dispensed medicine in secondary care to assess medicine concordance and inform individual patient's ongoing treatment. This supplements other data collected from clinical tests and patient-physician discussion. Development of a more efficient system to access the summary data is required before it is more widely used.

